# Peritoneal Ultrafiltration in the Long-Term Treatment of Chronic Heart Failure Refractory to Pharmacological Therapy

**DOI:** 10.3389/fphys.2019.00310

**Published:** 2019-03-28

**Authors:** Ewa Wojtaszek, Agnieszka Grzejszczak, Stanislaw Niemczyk, Jolanta Malyszko, Joanna Matuszkiewicz-Rowińska

**Affiliations:** ^1^Department of Nephrology, Dialysis and Internal Diseases, Medical University of Warsaw, Warsaw, Poland; ^2^Department of Nephrology and Internal Diseases, Military Institute of Medicine, Warsaw, Poland

**Keywords:** chronic progressive heart failure, cardiorenal syndrome, peritoneal ultrafiltration, icodextrin, patient survival, HF-related hospitalizations

## Abstract

**Introduction:**

Despite continuous improvement in the treatment, heart failure (HF) is a growing health problem and a major cause of mortality and morbidity in the world. There is some positive experience with the removal of the fluid excess via peritoneum in those patients, regardless of their renal function. The aim of this single center pilot study was to assess the efficacy of peritoneal ultra filtration (PUF) with a nightly 12-h exchange in the long-term treatment of refractory HF.

**Methods:**

The study included patients with chronic HF resistant to updated HF therapy (pharmacological and devices if applicable), who had experienced at least three hospitalizations due to HF during the preceding year and were disqualified from heart transplantation. All of them were treated with nightly 12-h 7.5% icodextrin exchange.

**Results:**

There were 15 patients (13 men), aged 72 ± 9 years, with charlson comorbidity index (CCI) 9 ± 1.2, NYHA class IV (11 patients) or III (4 patients), and eGFR 32 ± 11 ml/min/1.73m^2^. They were followed up for 24 ± 8 months (range 12–43, median 26 months). During the 1st year, all patients improved their NYHA functional class from 3.7 ± 0.5 to 2.6 ± 0.5; *P* = 0.0005, with stable (34.3 ± 12.4, and 35.6 ± 16.5%, respectively) left ventricular ejection fraction (LVEF), and inferior vena cava (IVC) diameter decreased from 27.8 ± 2.7 to 24.4 ± 3.4 mm; *P* = 0.09. Daily diuresis increased from 867 ± 413 to 1221 ± 680 ml; *P* = 0.25, while the dose of furosemide could be reduced from 620 ± 256 to 360 ± 110 mg/d; *P* = 0.0005, however, the kidney function deteriorated, with eGFR drop from 32 ± 11 to 25.6 ± 13 ml/min/1.73m^2^; *P* = 0.01). HF-related hospitalizations decreased from 8.9 ± 2.8 days/month to 1.5 ± 1.2 days/month (*P* = 0.003). Mechanical peritoneal dialysis complications occurred in five patients and infectious complications in four (peritonitis rate 1 per 72 patient-month). Patient survival was 93% at 1 year and 73% at 2 year. Technique survival was 100%.

**Conclusion:**

In patients with refractory HF, PUF with one overnight icodextrin exchange appears to be a promising therapeutic option as an adjunct to pharmacological management of those who are not transplant candidates. It should be emphasized that the treatment can have a great impact on the quality of life and the total costs of treating these patients.

## Introduction

Chronic progressive heart failure (HF) is an increasing public health problem of the 21st century and carries a poor prognosis. Paradoxically, improvement in diagnosis and treatment of ischemic heart disease and hypertension results in growing numbers of patients who survive acute cardiovascular events, at the expense of progressive HF, however, It’s been estimated that only in Europe more than 10 million people suffer from chronic HF, and approximately 5% of that population have reached the end-stage stadium of the disease, refractory to available therapies (Dickstein et al., 2008; Jessup et al., 2009). For the selected patients a device therapy, surgery and transplantation may be offered, however, many of them are not suitable for these invasive procedures, mainly due to age and coexisting diseases. Survival of such patients is less than 50% at 6 months, and the treatment with many hospitalizations constitutes a remarkable financial burden and confers suffering and a poor quality of life (Dickstein et al., 2008; Damman et al., 2009; Jessup et al., 2009).

The reduced cardiac output and fluid redistribution result in the decreased perfusion of other organs, including kidneys. The compensatory mechanisms such as activation of the sympathetic nervous system, renin-angiotensin-aldosterone (RAA) axis and arginine vasopressin lead to enhanced renal water and sodium retention in an effort to preserve glomerular filtration rate (GFR), but in the long-term these mechanisms are deleterious for both heart and kidneys (Mullens et al., 2009). The coexistence of cardiac and renal dysfunction induces a vicious circle that leads to an aggravation of both pathologies, chronic cardio-renal syndrome (CRS) development, and refractoriness to the treatment with decreased delivery of diuretics to their effector sites in the nephron (Ronco et al., 2008; Damman et al., 2009; Mullens et al., 2009). This spiral results in a further water and salt retention, further decline in cardiac output, and ultimately hypotension, with pulmonary edema and death (Ronco et al., 2008; Mullens et al., 2009).

There is a growing evidence that peritoneal dialysis (PD), with its flexibility in techniques, regimens and solutions, may be a feasible measure for patients with chronic CRS and refractory volume overload (Gotloib et al., 2005; Sánchez et al., 2010; Nuñez et al., 2012; François et al., 2015; Lu et al., 2015; Kazory, 2017). There are two patient groups with chronic CRS who could benefit from PD or PUF: patients with end-stage renal disease (ESRD) and those with preserved significant residual renal function. In the first group PD is needed as a method of not only water but also uremic toxins removal, while in the second one mainly or exclusively for a relief of refractory congestion.

The aim of this prospective single center pilot study was to assess the usefulness of PUF with one nightly 12-h icodextrin exchange in the long-term treatment of patients without ESRD with HF refractory to optimal medical therapy disqualified from heart transplantation.

## Materials and Methods

### Study Population

All consecutive patients with severe chronic HF (NYHA class III or IV) who fulfilled the inclusion criteria and were treated with PUF in our unit between January, 2005 and December, 2017 were enrolled into the study. The inclusion criteria were predefined as follows: (1) severe HF refractory to the optimal tolerable medical therapy, according to the current guidelines (maximal tolerable pharmacological therapy and implantable devices – ICD or CRT, if applicable); (2) contradictions for heart transplantation, (3) at least three hospitalizations for HF during last 12 months; (4) written informed consent. The exclusion criteria were: (1) inability of the patient or his assistant to cooperate; (2) presence of reversible causes of congestive HF (modifiable valvular heart disease, possible revascularization in coronary heart disease, hyperthyroidism, anemia, etc.); (3) infectious endocarditis; (4) presence of ESRD, (5) advanced malignant disease; (6) a history of myocardial infarction during last 90 days, myocarditis and pulmonary embolism during last 180 days.

The protocol was reviewed and approved by the Bioethical Committee of Medical University of Warsaw. All subjects gave written informed consent in accordance with the Declaration of Helsinki.

### Study Protocol

Double-cuff Tenckhoff catheter was implanted surgically under local or general anesthesia depending on an anesthetist’s decision. Four patients, before catheter implantation, required extracorporeal ultrafiltration (3–10 sessions, 1–2 L/day) due to massive water overload. All of the subjects were enrolled in a long-term program of PUF, initially with one nightly 12-h 7.5% icodextrin solution exchange. PUF was started with low-fill volume (1000 mL), which was gradually increased to 1500–2000 mL depending on individual patient’s tolerance.

The baseline clinical and laboratory parameters and number of hospitalizations for HF in the preceding year were recorded. During follow-up, patients were evaluated after one, 6 and 12 and 24 months after PUF initiation. Body weight, urine output, eGFR by MDRD formula, NYHA class, hospitalization days, PD complications and adverse events related to the treatment were recorded. Echocardiographic parameters were evaluated every 6 months.

### Statistical Analysis

All data were presented as mean ± standard deviation, median and interquartile range, and percentages, as appropriate. An analysis of differences between proportions was performed by means of Fisher’s exact test, and general linear model was used to assess any repeated measurements of the same variable. Peritonitis rates were calculated as episodes per patient-month at risk and hospitalization rates as days per month. Patient survival was analyzed using an actuarial method. A value of *P* < 0.05 was considered statistically significant. All calculations were performed using STATISTICA software package (version 12), StatSoft Poland.

## Results

Baseline clinical characteristics of the studied group are presented in the Table 1.

**Table 1:** Baseline clinical characteristics of the study population.

Parameter	Value
Age	72 ± 9 years
Male sex	87%
Charlson comorbidity index	9 ± 1.2
Type of cardiomyopathy (% of patients):	
Ischemic	47%
Valvular	33%
Restrictive	20%
PM or ICD or CRT (% of patients)	73% (28/36/36%)


*PM, pacemaker; ICD, implantable cardioverter-defibrillator; CRT, cardiac resynchronization therapy device.*

The time between Tenckhoff catheter placement and PUF commencement (break-in period) was 7.8 ± 4.0 days (median 8, range from 2 to 15 days). Slightly longer break-in period was noticed in patients with ascitic fluid drainage (8.8 ± 4.2 days, median 9). Nine (60%) patients were capable for self-care, the others needed assistance for PD procedures. The mean daily peritoneal ultrafiltration achieved with the technique was 927 ± 143 mL (median 1000 mL; 600–1200 mL).

The patients were followed up for 360 patient-months, mean 24 ± 8 months (median 26; 12–43 months). The treatment with only one overnight icodextrin exchange was continued for 13 ± 6 months (median 11; 7–33 months). The leading reason of the changes in PUF prescription such as increasing the number of manual exchanges or switching the patient to automated PD was the need for more intensive fluid and/or solutes removal. In three cases the causes were non-medical: two patients developed progressive dementia and needed assistance, and one patient due to loss of partner changed to intermittent PD, performed in the hospital three times a week.

During the first 6 months of the treatment a relief in congestion was obtained, with concomitant improvement in sensitivity to furosemide, hence in all patients a switch from intravenous into oral forms and the dose reduction (from 620 ± 256 to 277 ± 117 mg/d, *P* = 0.001) was possible (Table 2). Significant increase in urine output (from 867 ± 413 to 1807 ± 464 mL/d, *P* = 0.001), and a mild improvement of eGFR (32 ± 11 vs. 36.6 ± 13.8 mL/min/1.73m^2^, *P* = 0.59) were also noticed. However, during the next months the continuous deterioration of kidney function with urine output reduction was observed (Table 2).

**Table 2:** Kidney function, urine output, furosemide dose, and body weight during the follow up.

	Baseline	6 months	12 months	24 months	*P*-value
eGFR (mL/min/1.73m^2^)	32.0 ± 11.0	36.6 ± 13.8	25.6 ± 13.0	9.0 ± 13.0^§^	0.0001
Urine output (mL/day)	867 ± 413	1807 ± 464	1221 ± 680	480 ± 540^§^	0.0003
Furosemide dose (mg/d)	620 ± 256*	277 ± 117**	360 ± 110**	182 ± 192**	0.002
Body weight (kg)	78.5 ± 5.4	70.2 ± 5.3	69.0 ± 5.5	67.5 ± 5.3	0.0001


*eGFR, estimated glomerular filtration rate; ^∗^intravenous route of administration; ^∗∗^oral route of administration; §4 patients became anuric*.

During PUF almost all patients improved in their NYHA functional class by at least one. At 6 months there were 11 (73%) patients in NYHA class II and 4 (27%) in NYHA functional class III, at 12 months – 5 (33%) NYHA II, 9 (60%) NYHA III and 1 (7%) NYHA IV (Table 3). They also experienced an improvement, or at least stabilization in their LVEF, a decrease in IVC diameter, and a rise of MAP. This allowed for some pharmacological treatment modifications, particularly re-administer or further up-titration ACEi/ARB, mineralocorticoid receptor antagonists and hydrochlorothiazide to maximal tolerated doses (Table 3).

**Table 3:** Changes in echocardiographic parameters, MAP, NYHA class, all-cause, and HF-related hospitalizations during the follow-up.

	Baseline	6 months	12 months	24 months	*P*-value
LVEF (%)	34.3 ± 12.4	37.9 ± 10.8	35.6 ± 10.7	31.7 ± 9.8	0.1
IVC diameter (mm)	27.8 ± 2.7	22.4 ± 4.6	24.4 ± 3.4	26.8 ± 4.0	0.0003
MAP (mmHg)	58.8 ± 11.9	75.1 ± 11.0	76.0 ± 11.4	71.0 ± 11.2	0.0002
NYHA class (II/III/IV)	0/4/11	11/4/0	5/9/1	0/6/3	–
NYHA class (mean ± SD)	3.7 ± 0.46	2.3 ± 0.46	2.6 ± 0.5	3.3 ± 0.5	0.00006
Medications (% of patients):					
ACEi or ARB	27%	73%	55%	30%	
β-blockers	87%	100%	100%	90%	
MRI	20%	80%	73%	80%	
HCT	20%	53%	45%	22%	
All-cause hospitalizations	8.9 ± 2.8	1.2 ± 1.3	2.74 ± 1.4	2.57 ± 1.2	0.00003
(days/month)					
HF-related hospitalizations	8.9 ± 2.8	0.49 ± 0.8	1.48 ± 1.2	1.89 ± 0.9	0.00003
(days/month)					


*LVEF, left ventricular ejection fraction; IVC, inferior vena cava; MAP, mean arterial pressure; NYHA, New York heart association; ACEi, angiotensin converting enzyme inhibitor; ARB, angiotensin II receptor blocker; MRI, mineralocorticoid receptor antagonists (spironolactone or eplerenone); HCT, hydrochlorothiazide.*

### PUF Complications

The mechanical complications occurred in five patients. In three cases it was an inguinal hernia, in two of them the surgical repair was performed, with a moderate HF decompensation in a postoperative period surgery, demanding a temporary filling volume decrease in one patient and a switch to automated PD in the other. There were also two cases of exit-site leakage. In one patient with early leakage, the catheter was surgically re-transplanted, and after 3 days of break-in period PUF was restarted in a supine position. In the second patient a simple decrease in filling volume brought the resolution. To prevent infection in patients with exit-site leakage, antibiotic prophylaxis with cephalosporins was used.

Peritonitis was the only infectious complication. Overall five episodes were observed, for an average one episode per 72 patient-months. The mean time to first peritonitis episode was 12.7 ± 6.5 months (median 11 months), and no episode occurred in the 1st days after PUF start. The leading microbiologic etiology agent was Gram positive cocci (3 – coagulase-negative staphylococci, 1 – *Streptococcus salivarius*), and in one case *Escherichia coli*.

The mean hospitalization days related to PUF complications was 4.3 ± 8.3 days per month in the first, and 2.4 ± 4.9 days per month in the 2nd year of PUF.

In the study period, no episode of exit site/tunnel infection occurred. The typical procedures for the exit site care with povidone iodine were used in all patients (ointment with mupirocin is routinely not used in Poland).

Morbidity, expressed as all cause hospitalization days per month decreased significantly after start of PUF (Table 3). During the 1st year of PUF only one patient died (a sudden death), and three others died during the 2nd year (two from sepsis, one suddenly).

**FIGURE 1:**
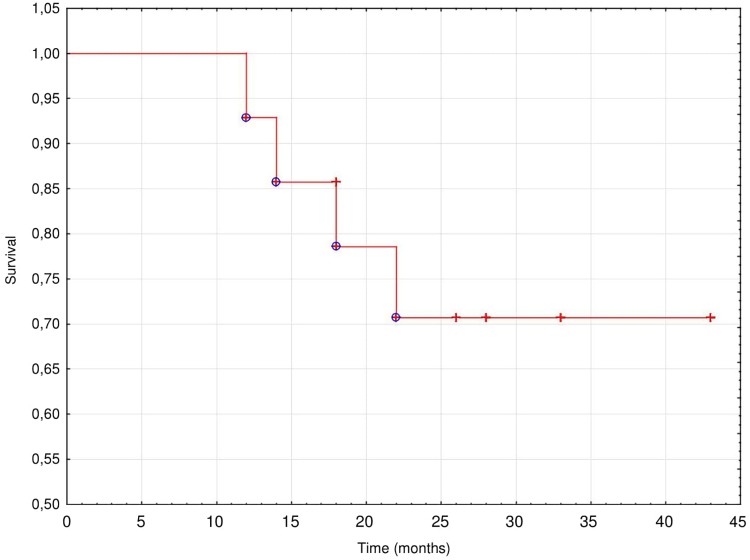
Cumulative actuarial patients’ survival.

At the end of observation 13 patients (87%) died, 9 (69%) of them survived > 24 months (mean 25.4 ± 8.0 months, (median 26 months) from the start of PUF. Most of patients died from a worsening of CHF (46%) or suddenly (31%), while 15% from sepsis and 8% from stroke. The technique survival censored for death was very good; 11 patients switched to full dose PD, three died while treated with PUF and one was lost to follow-up after 18 months of the treatment. The cumulative actuarial survival of the study population is presented on Figure 1.

## Discussion

The suggestion that PUF may offer clinically relevant benefits such as improvement in severe HF is not new. The first report of the successful use of PUF in this population was published in 1949, the second one in 1967 (Schneierson, 1949; Mailloux et al., 1967).

However, although many other studies have been performed since then, all of them were small, mainly of retrospective nature, done in heterogeneous populations, with patient inclusion criteria that were not always clear and with different treatment schedules. In this paper we present the results of an observational single study performed in a small but relatively homogeneous group of patients with refractory CHF and a long-term follow-up, where PUF with a one overnight icodextrin exchange was used as a rescue therapy. To make the group as homogeneous as possible, we excluded from the analysis patients treated with different PD solutions (dextrose based or mixed dextrose and icodextrin) as well as those with ESRD.

The major finding of our study was a spectacular, more than 80% decrease in hospitalization rates (for both the number of admissions and days spent in hospital), observed already after the 1st months of the treatment. This is in accordance with results of other studies, reporting at least 50% reduction in hospital admissions (Gotloib et al., 2005; Nakayama et al., 2010; Sotirakopoulos et al., 2011; Cnossen et al., 2012; Koch et al., 2012; Nuñez et al., 2012; Ruhi et al., 2012; Rizkallah et al., 2013; Bertoli et al., 2014) and with a recently published systematic review (Lu et al., 2015), where this was considered the most significant effect PUF. Such a marked decrease in the number of hospitalizations in our study may be partly due to the fact that in Poland it is not possible to administer iv furosemide in outpatient setting. Although all the patients were critically ill, with congestion depended to very high doses of iv diuretics, most of them started PUF without prior extracorporeal ultrafiltration. In other studies, extracorporeal ultrafiltration (or even hemodialysis) was used obligatorily or at least in selected patients to relief congestion, and some patients started PUF in an outpatient setting (Gotloib et al., 2005; Nakayama et al., 2010; Cnossen et al., 2012; Bertoli et al., 2014). Our observation suggests that most of the patients can safely and effectively initiate PUF without preceding extracorporeal ultrafiltration.

Peritoneal ultrafiltration was associated with a rapid, significant and long-term improvement in clinical status as demonstrated by the reduction in NYHA functional class, a feature observed in most published studies (Gotloib et al., 2005; Nakayama et al., 2010; Sánchez et al., 2010; Sotirakopoulos et al., 2011; Cnossen et al., 2012; Koch et al., 2012; Nuñez et al., 2012; Ruhi et al., 2012; Rizkallah et al., 2013; Bertoli et al., 2014). It was initially related with a significant, but transient improvement in LVEF. Indeed, the removal of excess fluid via ultrafiltration may improve cardiac output due to changes in the Frank-Starling curve, an increased left ventricular diastolic inflow, and an improvement in lung compliance (François et al., 2015; Kazory, 2017); however, only in some studies marked recovery of systolic left ventricular function was observed (Gotloib et al., 2005; Takane et al., 2006), while in others, the change was not significant (Ruhi et al., 2012; Rizkallah et al., 2013; Bertoli et al., 2014). This slow decrease in LVEF observed in our study was well compensated by PUF since the patients remained in better NYHA classes even after 24 months of the therapy, despite the progressive loss of RRF with urine output reduction. Our hypothesis is that an abrupt initial reduction in preload burden and ventricular end-diastolic volume with PUF leads to a significant rise in LVEF and the arterial pressure with better organs perfusion. This can explain a renal function improvement observed during the 1st months of the therapy. However, an increased afterload partially offsets the higher LVEF by rising the end-systolic volume.

In some reports pulmonary artery systolic pressure (PAPs) assessed by echocardiography was performed to evaluate the reduction of congestion after PUF/PD implementation (Nakayama et al., 2010; Sánchez et al., 2010; Koch et al., 2012; Nuñez et al., 2012; Bertoli et al., 2014). In our study, the improvement in volume status after the start of the PUF resulted in decrease in inferior vena cava diameter (IVCd). The measurement of IVCd with an assessment of its respiratory variability was chosen as a parameter to estimate the pressure in the right atrium, one of the non-invasive parameters in the diagnosis of pulmonary hypertension with an independent predictive value (Kalogeropoulos et al., 2014). In addition, it has been confirmed that increased IVC diameter itself identifies HF patients with adverse outcomes (Pellicori et al., 2013). The value of IVCd changes alone as a parameter in indirect assessment of right ventricle function and monitoring the response to PUF therapy warrants further evaluation.

The higher MAP allowed for the pharmacological HF treatment optimization, which in turn, together with a decongestion with PUF, resulted in a significant clinical improvement with the long-term reduction in the NYHA class. Unfortunately, the data on pharmacological treatment are not provided in the available studies.

Peritoneal ultrafilration therapy has also allowed for a restoration of sensitivity to diuretics and a rise in diuresis. It may be due to a better delivery of diuretics to their effector sites in the nephron along with intrarenal decongestion and a decreased stimulation of RAA axis and sympathetic nervous system (François et al., 2015; Kazory, 2017). Moreover, the controlled drainage of ascites might reduce intra-abdominal pressure, which has been demonstrated to improve renal function in HF (Mullens et al., 2009). The increase in daily diuresis comparable to that observed in our study was reported only by a few authors (Basile et al., 2009; Nakayama et al., 2010), others observed no change (Sánchez et al., 2010; Nuñez et al., 2012; Bertoli et al., 2014) or even its significant drop after 6 months of the treatment (Koch et al., 2012). It may be due to the selection bias, and other factors such as, for example, UF before the start of the PUF. Two studies, one prospective and one retrospective, have suggested that in non-ESRD patients PUF may preserve residual renal function (Nuñez et al., 2012; Bertoli et al., 2014). However, it could be due to a relatively short observation period, in both of them limited to 1 year. We observed a similar effect during the 1st year of the treatment in our patients, but later on their renal function deteriorated quickly and some of them became even anuric, requiring a full dose of dialysis.

Introduction of PUF resulted in effective ultrafiltration approximating 1000 mL/day, which together with augmented urine output resulted in a significant reduction in body weight, similar to described in some (Sotirakopoulos et al., 2011; Cnossen et al., 2012; Nuñez et al., 2012), but not all studies (Nakayama et al., 2010; Sánchez et al., 2010; Bertoli et al., 2014). However, interpreting this finding is difficult. While in the 1st months of the treatment it apparently was related to relief of congestion and improved volume status, in the later period it could as well indicate a progressive malnutrition typical for severe chronic diseases. Therefore, more objective assessment of body composition such as bioimpedance should be employed, as in the study by Gotloib et al. (2005).

It should be stressed that the low all-cause hospitalizations rates (including those related to comorbid conditions and PUF itself) persisted during the entire follow-up, despite a gradual worsening of HF and renal function. One third of studied patients died suddenly, but the quality of their last year of life, spent at home and with less clinical symptoms, seemed to be much better, although not measured by any standardized questionnaire. This huge reduction in hospital admissions, despite the cost of PUF, may also have a marked financial aspect, as shown by Sánchez et al. (2010).

In our study 2-year patient survival rate of 73% was similar, even slightly better than that reported in other studies (Sánchez et al., 2010; Rizkallah et al., 2013; Bertoli et al., 2014). Taking into account the poor prognosis of these patients resulting from HF and a high comorbidity burden our data give some hope for an improvement not only in the quality of life but also in survival compared with patients receiving conservative therapies.

We are fully aware of the limitations of our study, being a pilot, single-center, retrospective and observational study with relatively small sample size, therefore the results should be interpreted with caution. However, single center character allows implementation of consistent, standardized approach to the management and surveillance during the treatment of PUF. Moreover, we included all the consecutive patients and followed them for a median of 26 months, much longer than in other studies (Sánchez et al., 2010; Cnossen et al., 2012; Koch et al., 2012; Nuñez et al., 2012; Ruhi et al., 2012). It should be also stressed that to perform study on PD patients always is associated with problems with sample size due to prevalence of this modality among dialyzed patients.

## Conclusion

Peritoneal ultrafiltration appears to be a reasonable strategy for the treatment of refractory HF as a rescue therapy in patients who are not eligible for heart transplantation when the optimal medical treatment failed. It offers a relevant clinical benefit due to a significant reduction in hospitalization rate, better quality of life, and perhaps even some survival advantage. With the progression of CKD incremental PD can be implemented as a self-care or an assisted procedure. There are many questions that remain unanswered such as when PUF should be considered. Perhaps not randomized, but larger observational clinical trials should be designed and performed to provide more information and establish the best protocol for the management of refractory HF in patients with concomitant chronic kidney disease. Cost-benefit analyses and reimbursement policies should also be implemented.

## Author Contributions

EW conceived and designed the study, collected of the data, performed the statistical analysis, wrote the manuscript, and prepared its final version. SN designed the study. AG collected the data and analyzed the data. JM-R conceived and designed the study, wrote the manuscript, and prepared its final version. JM wrote the manuscript and prepared its final version. All the authors approved the final version of the manuscript.

## Conflict of Interest Statement

The authors declare that the research was conducted in the absence of any commercial or financial relationships that could be construed as a potential conflict of interest.
